# EBV-Driven NK/T-Cell Lymphoproliferative Disorders: Clinical Diversity and Molecular Insights

**DOI:** 10.3390/lymphatics4010007

**Published:** 2026-01-26

**Authors:** Aleksander Luniewski, Sahil Chaudhary, Adam Goldfarb, Ifeyinwa E. Obiorah

**Affiliations:** Section of Hematopathology, Department of Pathology, University of Virginia Health, 1215 Lee Street, Charlottesville, VA 22903, USA

**Keywords:** EBV, NK/T cell, lymphoma, lymphoproliferative disorder, molecular

## Abstract

The World Health Organization (WHO) and International Consensus Classification (ICC) systems have classified EBV-positive NK/T-cell neoplasms in adults and EBV-positive T/NK-cell lymphoid lymphoproliferative disorders (LPD) in children. Recent molecular profiling techniques have revealed the pathogenesis of these disorders, showing interactions among EBV-encoded proteins, host immune responses, and genetic alterations. Extranodal NK/T-cell lymphoma (ENKTL) shows molecular diversity, with various subtypes (TSIM, MB, and HEA) identified through a multiomics approach. Aggressive NK-cell leukemia (ANKL) has mutations in JAK/STAT, epigenetic regulators, and *TP53* pathways. EBV-positive nodal T- and NK-cell lymphoma (ENTNKL) is a new entity, distinguished by primary nodal presentation and a unique molecular profile. Severe mosquito bite allergy (SMBA), hydroa vacciniforme lymphoproliferative disorder (HVLPD), and systemic chronic active EBV disease (CAEBV) are rare childhood EBV-driven LPDs defined by clinico-pathologic criteria, with largely unexplored genomic landscapes. Studies of CAEBV samples have found ENKTL-like driver mutations, including *DDX3X* and *KMT2D*, in EBV-infected NK/T cells, while *KMT2D* and chromatin modifier mutations were common in HVLPD. Comprehensive molecular sequencing of SMBA and Systemic EBV-positive T-cell lymphoma of childhood remains lacking. These findings suggest all EBV+ NK/T-cell LPDs exist on a biological continuum of viral oncogenesis. The integration of clinical, pathological, and molecular information aims to create a more accurate classification system, enabling better risk evaluation and tailored treatment strategies for patients with these complex disorders.

## 1. Introduction

During the initial infection, Epstein–Barr virus (EBV) targets B cells and epithelial cells, with the potential to also infect certain T/natural killer (NK) cells [1]. In some cases, due to inadequate presentation by specific human leukocyte antigens or genetic factors, EBV-infected NK/T cells manage to escape the host’s immune response and persist [2]. EBV-associated NK/T-cell lymphoproliferative disorders (LPD) are characterized by the proliferation of EBV-infected NK/T cells, exhibiting a range of clinical manifestations, from indolent to highly aggressive forms [3]. They have the potential to affect multiple organs and tissues throughout the body. World Health Organization (WHO) [4] and international consensus classification (ICC) [5] systems have categorized EBV-positive NK/T-cell neoplasms for adults and EBV-positive NK/T-cell lymphoid proliferations and lymphomas for children (Table 1). In children, EBV–NK/T disorders include severe mosquito-bite allergy (SMBA), hydroa vacciniforme lymphoproliferative disorder (HVLPD), systemic chronic active EBV disease (CAEBV), and systemic EBV-positive T-cell lymphoma of childhood (STCLC). In adults, the primary EBV-associated conditions are extranodal NK/T-cell lymphoma (ENKTL), aggressive NK-cell leukemia (ANKL), and, more recently, EBV-positive nodal T- and NK-cell lymphoma [4], otherwise known as primary nodal EBV–positive T-cell/NK-cell lymphoma [5]. EBV facilitates lymphomagenesis through its latent gene products, limiting their expression to evade immune detection. EBV-positive NK/T LPDs are typically associated with a Latency I/II transcriptional program, which includes EBNA-1, LMP-1, LMP-2a&b, and miRNAs, although a subset of cases do not express LMP-1 [6,7]. The precise mechanisms by which EBV latency I/II infection in NK/T cells lead to LPD development remain unclear, but EBV can interact with the host cell through various pathways. Recent advancements in molecular profiling techniques have shed light on the pathogenesis and progression of EBV-associated NK/T-cell lymphoproliferative disorders, which involve intricate interactions among EBV-encoded proteins, the host’s immune response, and genetic changes in the affected cells. These discoveries have not only deepened our understanding of the disease’s biology but have also facilitated the development of targeted treatments and enhanced diagnostic methods. Integrating clinical, pathological, and molecular information aims to create a more accurate classification system, enabling better risk evaluation and tailored treatment strategies for patients with these complex disorders.

## 2. Extranodal NK/T-Cell Lymphoma

**Figure 1.**
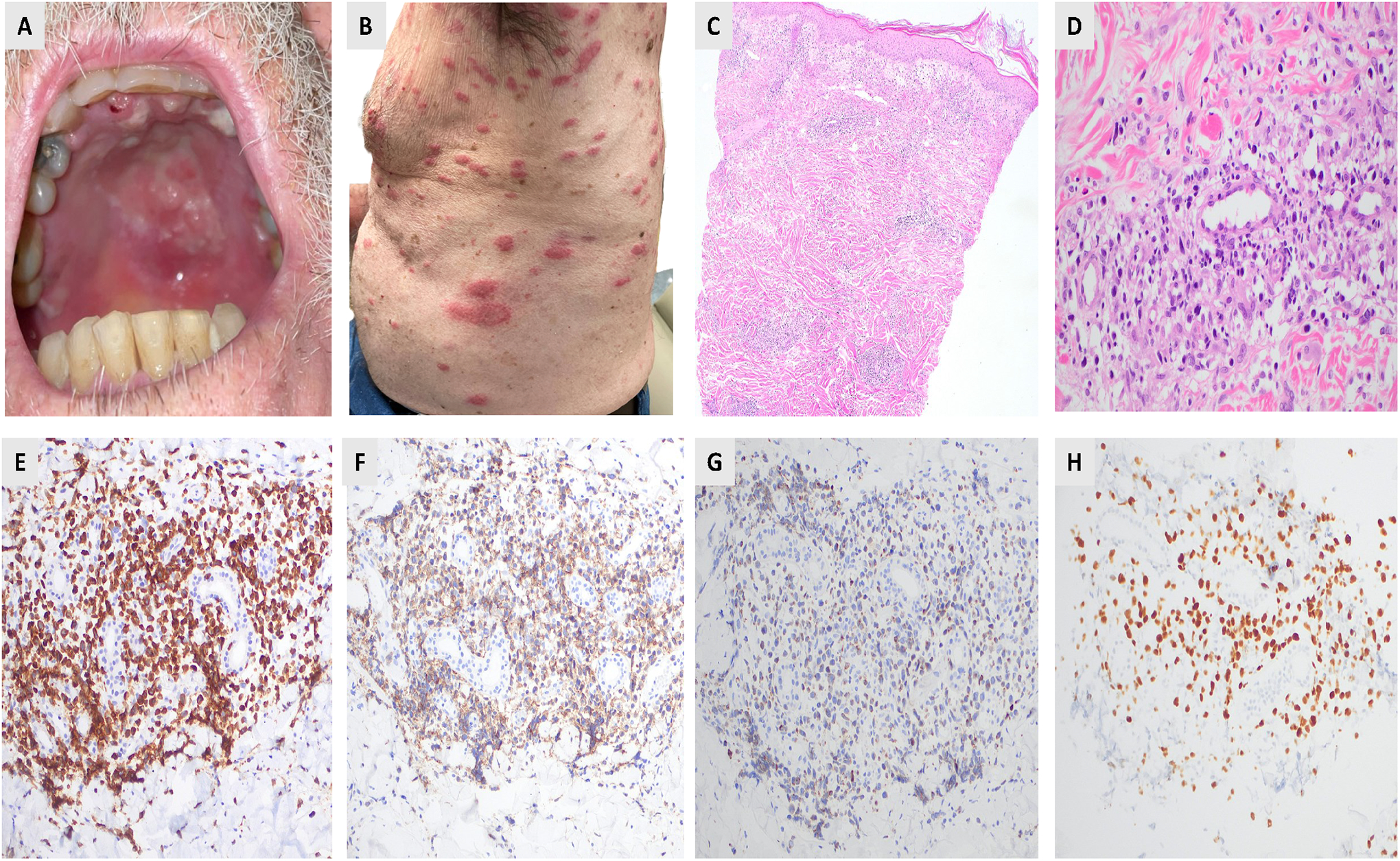
Clinical and pathologic presentation of the extranodal NK/T-cell lymphoma. Clinical photographs showing mucosal lesions in the oral cavity (**A**) and generalized papular and nodular cutaneous lesions (**B**). Skin punch biopsy from the back, revealing a patchy dermal infiltrate with a perivascular distribution (**C**, H&E stain, 10×). The atypical lymphocytic infiltrate exhibits an angiocentric pattern and consists of neoplastic lymphoid cells of medium to large size, interspersed with histiocytes and small lymphoid cells. (**D**, H&E stain, 40×). Immunohistochemistry (IHC) of skin biopsy confirm positive expression of (**E**) CD3, (**F**) CD56, (**G**) perforin (IHC, 40× each), and (**H**) EBV RNA (RNAscope, 40×).

ENKTL was classified as extranodal NK/T-cell lymphoma, nasal type, in the WHO 2016 classification [8]. Although the term “nasal type” was omitted in the WHO fifth edition, the ICC classification continues to retain it [5], to emphasize that cases occurring outside the nasal area exhibit identical morphological characteristics [9]. ENKTL is significantly more prevalent among Asians and Latin Americans, most common in the fourth and fifth decades of life [10,11]. Based on the primary tumor site, ENKTLs are classified as nasal and non-nasal subtypes (Figure 1A). In clinical settings, patients frequently exhibit destructive lesions in the midface, such as nasal blockage and nosebleeds, or they may have widespread disease affecting the skin (Figure 1B), gastrointestinal tract, or testis or other extranodal sites [12]. Histologically, ENKTL exhibits an angiocentric (Figure 1C,D) and angiodestructive infiltrate of atypical lymphoid cells, interspersed with plasma cells, histiocytes, and eosinophils [13]. Necrosis may be extensive, indicating vascular destruction. The neoplastic cells range from small to medium-sized forms with irregular nuclei to larger cells, with frequent mitoses. Immunophenotypically, as illustrated in Figure 1E–H, the tumor cells predominantly exhibit characteristics of cytotoxic NK cells, with a lesser presence of T-cell lineage. These cells frequently express cytoplasmic CD3 and CD56. Additionally, EBV is detected in nearly all cases. Recent studies have revealed that extranodal NK/T-cell lymphoma (ENKTL) is molecularly diverse, with distinct subtypes identified through comprehensive genomic, transcriptomic, and epigenetic analyses. Xiong and colleagues [14] described three molecular subtypes, namely MB, TSIM, and HEA, which not only represent the underlying biological differences but also have associations with clinical outcomes (Table 2). TSIM subtype (tumor suppressor–immune modulator) features JAK/STAT pathway alterations, PD-L1/PD-L2 amplification, and frequent 6q21 deletions affecting tumor suppressors like PRDM1. This subtype shows immune modulation and better prognosis with potential immunotherapy sensitivity. MB (MGA-BRDT) is defined by MGA mutations, epigenetic regulator alterations, and MYC-related pathways, showing aggressive behavior and poor outcomes. HEA (HDAC9-EP300-ARID1A) has mutations in epigenetic regulators, affecting chromatin remodeling and transcription, with intermediate prognosis and potential for epigenetic-targeted therapies. ENKTL is historically managed with conventional chemotherapy, especially L-Asparaginase-based therapy, SMILE (dexamethasone, methotrexate, ifosfamide, L-asparaginase, and etoposide) regimen [15]. Advances in understanding its molecular pathogenesis have expanded potential treatment options to include targeted therapies such as brentuximab vedotin, immune checkpoint inhibitors [16], monoclonal antibodies [17], epigenetic modulators, and novel pathway-directed agents [18], representing a significant shift toward precision and immune-based strategies for refractory and advanced disease.

## 3. Aggressive NK-Cell Leukemia

Aggressive NK-cell leukemia (ANKL) is a rare NK-cell malignancy, involving younger adults with a median age of 20–40 years and shows geographic predilection for East Asia and Central/South America [19]. The disease is strongly associated with EBV infection, with most cases showing EBV positivity through EBER hybridization. Patients often present with acute systemic illness, including fever, hepatosplenomegaly, cytopenias, liver dysfunction, disseminated intravascular coagulation (DIC), and hemophagocytic lymphohistiocytosis (HLH). The classic ANKL disease may progress rapidly, causing multiorgan failure, with a median survival of under 2–3 months without therapy [20]. In contrast, a subacute form of ANKL, characterized by an extended prodromal period and generally improved survival rates, has been described [21]. The peripheral blood, bone marrow, liver, and spleen are the most frequently affected areas, although any organ can be involved [21]. Biopsies show varying infiltrative growth patterns, ranging from subtle with a sinusoidal pattern to a more diffuse infiltrate of atypical lymphoid cells with irregular nuclei, coarse chromatin, and frequent mitoses and apoptosis. Angiocentric patterns and hemophagocytosis are common, with necrosis in extranodal sites. The neoplastic cells show CD2+, cytoplasmic CD3ε+, surface CD3–, CD56+, and cytotoxic molecule expression, typically negative for CD4 and CD8, with variable CD16 expression [22,23]. The TCR genes maintain their germline configuration because the tumor cells are derived from the NK-cell lineage. Sequencing studies reveal mutations in JAK/STAT pathway (*JAK3, STAT3, STAT5B*), epigenetic regulators (*TET2, KMT2D, CREBBP*), *TP53* [24]*, DDX3X* and other signaling pathways including (*PIK3CB, NFKB1, NFKBIA, MAP3K13*) [23–25]. Gene mutation patterns were similar between classic and subacute ANKL subtypes, while *TP53* gene mutations were enriched in classic ANKL patients [19]. These alterations combine with EBV-driven oncogenesis to promote immune evasion. Conventional anthracycline-based treatments are ineffective due to multidrug resistance. L-asparaginase–based regimens show better response rates, though relapses are common [26,27]. Allogeneic hematopoietic stem cell transplantation offers the only chance for durable survival [28,29]. Novel approaches include JAK inhibitors, immune checkpoint inhibitors, and EBV-targeted therapies, though prognosis remains poor [30].

## 4. EBV-Positive Nodal T- and NK-Cell Lymphoma

EBV-positive nodal T- and NK-cell lymphoma (ENTNKL) was first identified as a distinct diagnostic category in the 2022 WHO fifth edition [4] and as a provisional entity by the ICC as primary nodal EBV-positive T-cell/ NK-cell lymphoma [5]. It is distinguished from ENKTL due to its primary nodal presentation, unique clinical course, and molecular profile, despite both being associated with EBV [31,32]. Patients typically present with generalized lymphadenopathy, often without the nasal or extranodal involvement characteristic of ENKTL [33]. Occasionally, it might also affect a small number of extranodal sites. Systemic symptoms such as fever, weight loss, and night sweats are prevalent, and advanced-stage disease at diagnosis is common [33]. This condition predominantly affects older adults (median age of 61–64 years), with cases primarily reported in East Asia. The nodal architecture is generally effaced by a diffuse infiltrate of medium- to large-sized atypical lymphoid cells, occasionally exhibiting centroblastic or anaplastic morphology [33,34]. Angiocentricity and angioinvasion are less pronounced than in ENKTL. The neoplastic cells are EBV-positive by in situ hybridization. They often express CD3, CD2, CD8, CD56, cytotoxic markers (TIA-1, granzyme B, perforin), and are typically CD5 and CD4 negative. PD-1 and TFH markers are absent, excluding TFH lymphomas. Unlike ENKTL, this lymphoma is derived from T cells in >80% of cases, shown by T-cell receptor expression and TR gene rearrangement [32]. Wai and colleagues [35] found ENTNKL showed mutations of *TET2, PIK3CD* and *STAT3*, with microsatellite stability but lower genomic instability than ENKTL and peripheral T-cell lymphoma, not otherwise specified (PTCL, NOS). Nevertheless, ENTNKL demonstrated superior overall survival compared to cytotoxic PTCL, NOS, but not when compared to non-cytotoxic PTCL, NOS. ENTNKL was associated with 14q11.2 loss, increased PD-L1 and T-cell genes (CD2, CD8), and decreased CD56, consistent with CD8+/CD56- immunophenotype [36]. These alterations partially overlap with ENKTL but underscore a nodal-specific biology. ENTNKL lacks standard treatment, with poorer survival than ENKTL and PTCL, NOS [32,35]. Similar to ENKTL, anthracycline-based chemotherapy is ineffective. Further investigations are needed to examine the effects of L-asparaginase-based therapy and novel targeted therapies on ENTNKL.

## 5. Systemic Chronic Active EBV Disease

Systemic chronic active EBV disease (CAEBV) is a progressive disorder lasting ≥ 3 months with increased EBV DNA levels in blood and organ infiltration by EBV-infected lymphocytes without immunodeficiency [37]. Previously called CAEBV infection, the term “CAEBV disease” is now preferred because most adults are chronically infected with EBV [5]. While it formerly included T, NK, or B cells, CAEBV should only include T- or NK-cell disease, as B-cell cases often indicate primary immunodeficiency. CAEBV usually affects otherwise apparently immunocompetent children and young adults from Eastern Asia and South and Central America. Clinically, it presents as a prolonged “mononucleosis-like” illness (fever, malaise) with hepatosplenomegaly and lymphadenopathy. Patients commonly develop high-grade systemic inflammation—including recurrent or chronic fevers, pancytopenia, hepatitis, gastrointestinal ulceration, and pneumonitis—and often life-threatening complications such as hemophagocytic lymphohistiocytosis (HLH), vasculitis (including coronary aneurysms) and liver failure [37,38]. Cutaneous EBV-LPD manifestations such as HVLPD and SMBA are occasionally observed in pediatric cases. While some patients exhibit severe symptoms that deteriorate rapidly, others remain stable without the need for treatment. A subset of patients eventually develops ANKL or ENKTL. Biopsied tissues show small to medium lymphocytes infiltrating these organs without overt cytologic atypia [39]. Lymph nodes display paracortical hyperplasia and polymorphous lymphocytic proliferation with inflammatory cells. The lymphoid cells are of either cytotoxic T-cell or NK-cell lineage, as indicated by the expression of sCD3, CD56, and T-cell receptor proteins, as well as TR gene rearrangements [40]. Molecular analysis reveals that the EBV genomes in CAEBV cells most often exhibit a type II latency pattern [39,40]. Recent genomic studies have demonstrated that mutations in *DDX3X, KMT2D, BCOR/BCORL1, KDM6A*, and *TET2* are frequent in CAEBV [41]. Notably, *DDX3X* mutations, which are also present in ENKTL, serve as the primary driver mutation. A potential transformation may occur through the acquisition of additional somatic mutations, leading to clonal evolution. Furthermore, distinct large deletions in the EBV genome, often occurring in the BART miRNA clusters and lytic genes, have been identified in CAEBV. These deletions may disrupt viral latency and encourage cellular proliferation. Wang et al. [42] found that EBV infects the hematopoietic system, including lymphoid and myeloid lineages and hematopoietic stem cells (HSCs) in CAEBV patients. HSC infection causes systemic inflammation through proinflammatory cytokines, which are elevated in CAEBV patients [43]. After HSC transplantation (HSCT), EBV-infected cells are eliminated [42,44]. The prognosis is generally poor, and no universally effective pharmacological treatment exists for CAEBV. While antiviral agents or chemotherapy may temporarily manage the disease, they seldom achieve complete eradication. Evidence-based practice standards concur that HSCT is the only treatment with curative potential [45].

## 6. Hydroa Vacciniforme Lymphoproliferative Disorder

**Figure 2.**
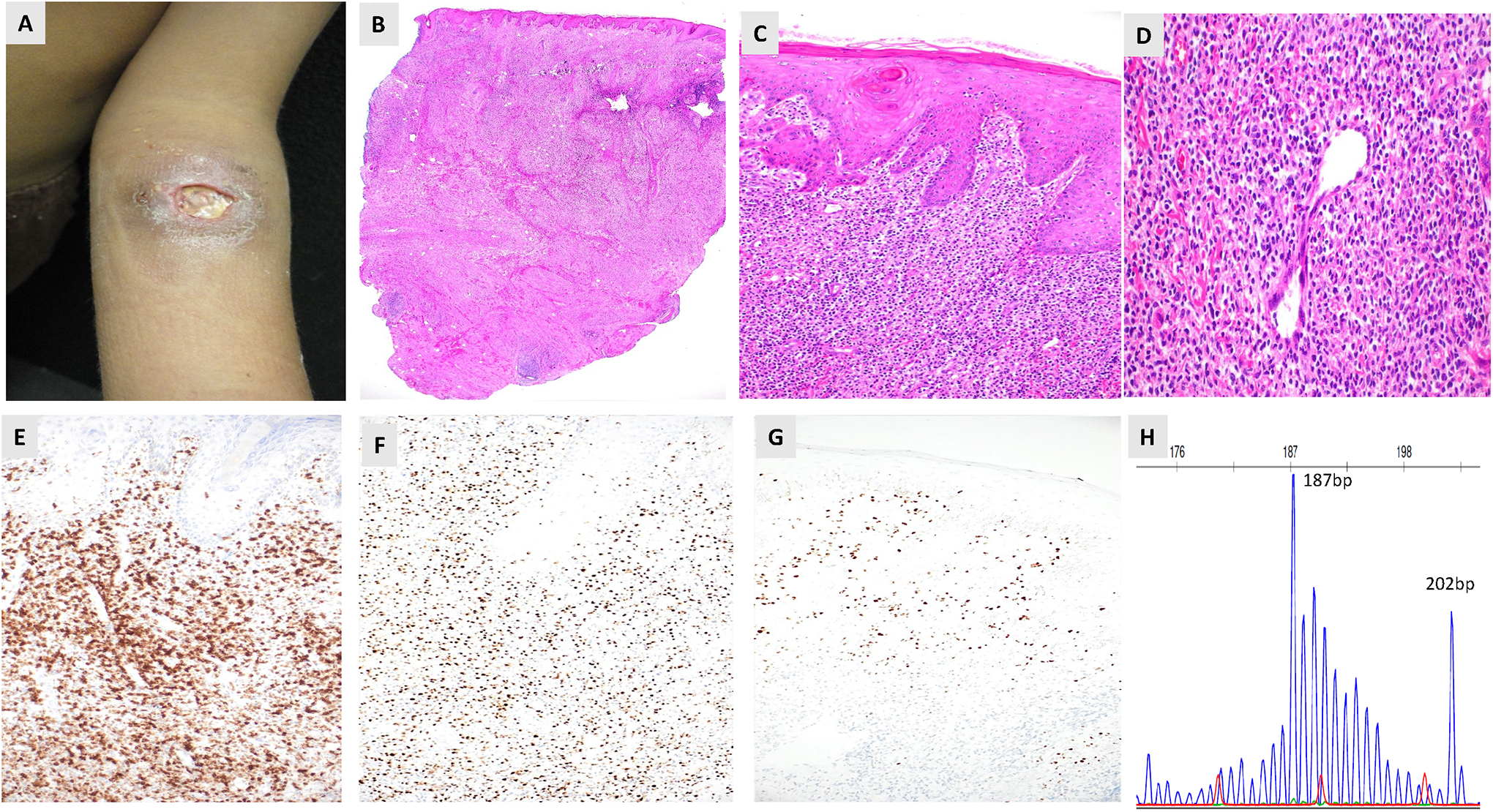
Clinical and pathologic presentation of the hydroa vacciniforme lymphoproliferative disorder. (**A**) Clinical photograph of a skin lesion in the left upper extremity characterized by erythematous vesicles, papules, crusts, and scars. (**B**) Histologic section demonstrates an infiltrate mainly in the dermis that extends into the subcutaneous fat (H&E stain, 5×). (**C**–**D**) The lymphocytes were mostly small to medium-sized with moderate cytologic atypia surrounding the blood vessel (H&E stain, 40×). Immunohistochemistry (IHC) of skin biopsy demonstrates positive expression of (**E**) CD3, (**F**) CD8 (IHC, 40× each) and (**G**) EBV RNA (RNAscope, 40×). (**H**) The TCR gene rearrangement analysis demonstrates clonal peaks at 187 bp and 202 bp in the skin biopsy.

**Figure 3.**
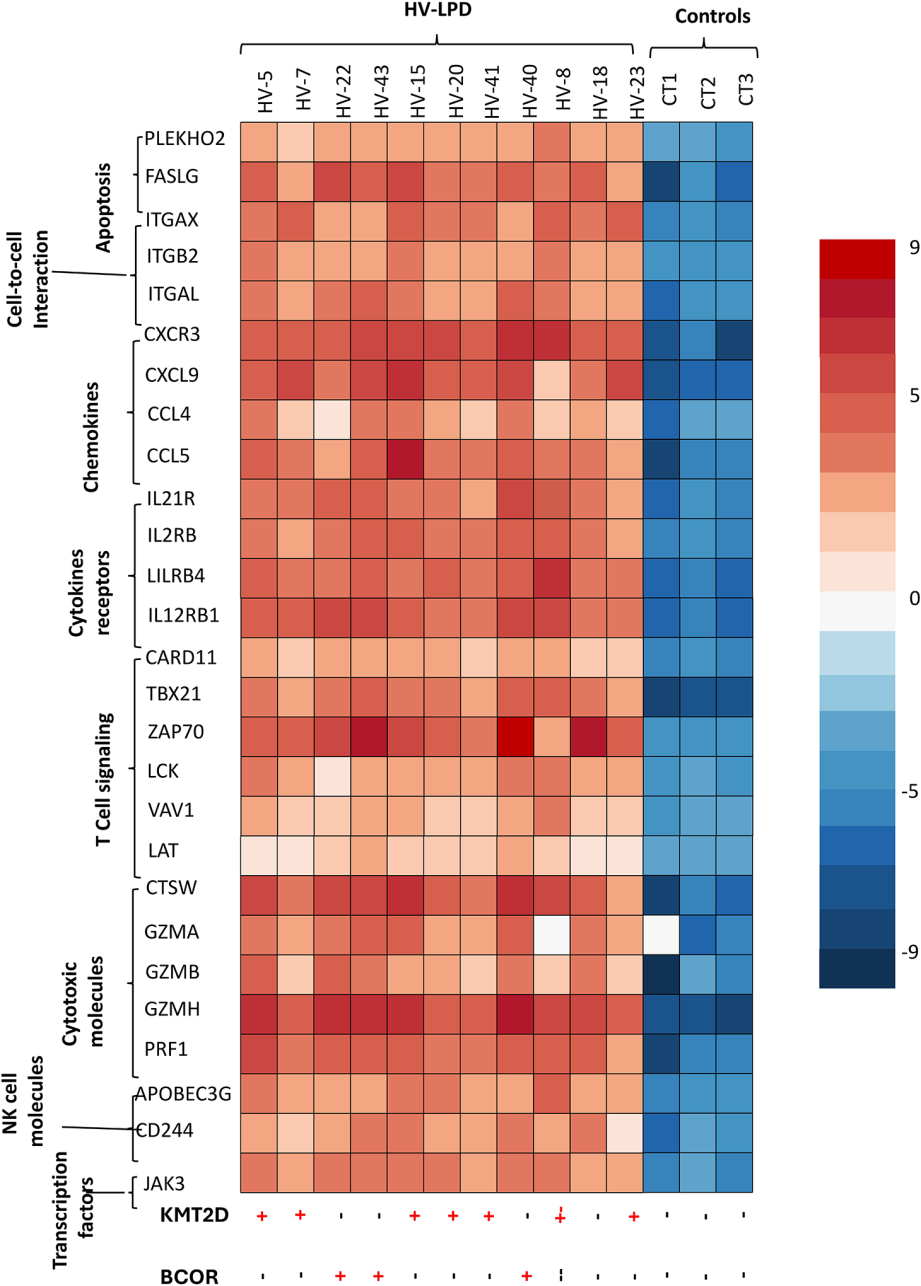
Differential gene expression in select HVLPD patients with KMT2D and BCOR alterations. The heat map shows log-fold changes in expression, with red representing increased expression and blue representing decreased expression levels of different pathway-related genes observed in HVLPD patients (*p* < 0.05). The scale bar represents the level of gene expression. Cases with KMT2D alterations and BCOR mutations are shown at the bottom of the graph. Reproduced from Lewinski et al [52], with permission from Elsevier, Mendeley Data, V1, 2025. Licensed under the Creative Commons Attribution 4.0 International License (CC BY 4.0).

Hydroa vacciniforme (HV) lymphoproliferative disorder (HVLPD) is a chronic Epstein–Barr virus (EBV)-positive lymphoproliferative disorder of childhood. HVLPD is a cutaneous form of CAEBV disease ranging from classic photodermatitis to systemic form with extensive skin lesions and multiorgan involvement. This disorder was initially described as benign photodermatosis in western countries, resolving in adolescence or young adulthood [46]. A more severe form was discovered in children from Asia, Latin America, and Mexico, showing increased lymphoma development [47]. These lesions present with facial edema, vesicles, ulcers, and scarring in sun-exposed and nonexposed areas [48] (Figure 2A). Systemic symptoms, including fever, hepatosplenomegaly, and lymphadenopathy, were termed “severe” HV to differentiate from benign “classic” HV described in the western world. Systemic HVLPD lesions are not triggered by sun exposure [49]. Disease progression manifests through ineffective photoprotection, facial swelling, and systemic complications. Both forms are recognized as HVLPD by the new WHO and ICC classification systems. Histological examination reveals intraepidermal vesicles with lymphocyte infiltration in the dermis, predominantly in periadnexal and perivascular regions (Figure 2B–D). The lymphocytes are small to medium-sized, showing mild atypia and irregular nuclei, interspersed with inflammatory cells [49]. HVLPD manifests as T or natural killer (NK) cell lineage, with most cases being T-cell lineage (Figure 2E–H). The lymphoid cells show a CD8+ cytotoxic phenotype, although CD4+ or CD4+CD8+ phenotypes have been documented. The mutation landscape of HVLPD is poorly understood. Cohen and colleagues [50] found no mutations but demonstrated increased IFN-γ expression and upregulation of chemokine genes in a Caucasian patient with HVLPD. Xie et al. [51] detected *STAT3, IKBKB, ELF3, CHD7* and *KMT2D* mutations in 5 Chinese patients with HV-LPD, but none were recurrent. Luniewski and colleagues [52] performed the first genome-wide comprehensive study on 28 patients with HVLPD from Latin America and Mexico. Analysis revealed somatic mutations, with epigenetic regulator KMT2D being the most frequently mutated gene (28%), followed by CREBBP (21.4%), and BCOR (14.3%). Inactivation of tumor suppressor genes (TSG), including ATM (14.3%), MLH1 (10.7%) and BARD1 (10.7%) were observed. Copy number analysis revealed deletions involving TSGs, MAP2K4 (50%), SMARCB1 (36%), and FANCA (36%). Differential gene expression analysis identified significant gene overexpression in cytokine signaling, NK/T-cell activity, and JAK signaling. Analysis of serial samples from three patients at various stages of HVLPD revealed an increase in EBV tumor content and mutational burden in chromatic modifier genes. Similar to CAEBV, there was a reduced acquisition of somatic driver mutations, such as those in the JAK/STAT and TP53 pathways. Systemic HVLPD and CAEBV infection both exhibit a chronic progression within the context of CAEBV disease, leading to systemic symptoms and the potential development of lymphomas. HVLPD is characterized by an upregulation of genes associated with cytokine signaling and the activity of T and NK cells (Figure 3), paralleling observations in CAEBV patients [42,43]. The overall observations suggest that systemic HVLPD in Latin America or Asia, as well as CAEBV, may represent a transitional phase between classic HVLPD and the more aggressive forms of ANKTL and ENKTL. Both disease entities are associated with generally poor prognoses, with HSCT being the sole curative treatment option [42,50].

## 7. Severe Mosquito Bite Allergy

Severe mosquito bite allergy (SMBA) is a rare lymphoproliferative disorder driven by Epstein–Barr virus (EBV) and involving natural killer (NK) cells, and occasionally T cells. This condition has traditionally been referred to as hypersensitivity to mosquito bite or an exaggerated insect bite reaction. Unlike typical mosquito allergies, CD4+ T cells in the skin respond to mosquito saliva, triggering the reactivation of latent EBV in NK cells, which results in inflammation [53]. Elevated levels of interleukin 13 and immunoglobulin E are observed. The condition is now recognized as a cutaneous form of CAEBV of the T/NK-cell type by the 2022 WHO and ICC classifications. In the 2022 World Health Organization classification, SMBA is explicitly categorized among EBV-positive T/NK-cell proliferations in childhood. SMBA predominantly affects children, especially under the age of 10 years [54]. Most cases have been reported from East Asia, Latin America, or Mexico, with no apparent gender bias. The primary clinical features include necrotizing skin lesions following mosquito bites, often presenting as large, erythematous, indurated patches that may blister or ulcerate, and may be accompanied by transient systemic symptoms [54,55]. High fever, chills, malaise, lymphadenopathy, and elevated liver enzymes may follow a bite. Between episodes, patients are generally asymptomatic, although laboratory tests reveal chronic inflammation, characterized by markedly elevated serum IgE, high EBV viral DNA load in the blood, and persistent NK-cell lymphocytosis [56]. Some patients show skin manifestations resembling HVLPD. Over time, some patients may develop hemophagocytic lymphohistiocytosis (HLH) or progress to overt ENKTL or ANKL [56,57]. SMBA shows epidermal necrosis and ulceration. Small and large lymphoid cells and histiocytes infiltrate the upper dermis and subcutaneous tissue, with EBV-positive cells. Advanced lesions display angiocentric and angiodestructive growth [54]. Most cases demonstrate an NK-cell immunophenotype, although a subset shows a T-cell phenotype. The NK cells lack surface CD3 and T-cell receptor positivity, but show expression for cytoplasmic CD3ε, CD16, CD56, and cytotoxic markers such as TIA-1 and granzyme B. Molecular pathogenesis of SMBA is poorly characterized, and the genomic landscape remains largely unexplored (Table 3). EBV-encoded gene expression, including expression of BZLF1, has been observed in SMBA [57] and correlates with a poor prognosis.

## 8. Systemic EBV-Positive T-Cell Lymphoma of Childhood

Systemic EBV-positive T-cell lymphoma of childhood (STCLC) affects children and young adults, particularly in Asia, often following primary acute EBV infection [58]. Patients present with acute febrile illness, hepatosplenomegaly, lymphadenopathy, skin rash and cytopenias, frequently showing features of hemophagocytic lymphohistiocytosis (HLH [59]. The clinical course is fulminant, with multiorgan failure and rapid progression to death within days to weeks of onset [59,60]. Tissue biopsies reveal a subtle to diffuse infiltrate of EBV-positive atypical lymphoid cells that efface normal architecture. Cells are frequently small and lack cytological atypia, but they can also be medium to large, featuring irregular nuclei, apoptotic debris, and necrotic areas [61]. Hemophagocytosis frequently occurs in the bone marrow and liver, often accompanied by a sinusoidal infiltrate. The neoplastic cells express T-cell markers: CD2 and CD3, TIA-1, granzyme B, and perforin, confirming a cytotoxic T-cell phenotype. In most instances, secondary cases due to an acute primary EBV infection are CD8+, whereas those associated with systemic CAEBV disease tend to be CD4+ [62]. EBV-encoded RNA (EBER) is strongly positive by in situ hybridization. Frequent T-cell receptor (TCR) clonality reflects clonal cytotoxic T-cell expansion. The mutational landscape is less characterized compared with ENKTL. While specific chromosomal abnormalities have not been discovered in SCTCL, its gene expression profiling closely resembles that of ENKTL, marked by the overexpression of p53, survivin, and EZH2 [63]. SCTCL is associated with a very poor prognosis, characterized by uniform morphology and elevated levels of cyclin E2 and Ki67, which are recognized as indicators of unfavorable outcomes [64]. Conventional chemotherapy is generally ineffective. Supportive measures, including HLH-directed therapy, may be administered and may be followed by allogeneic HSCT [10,59].

## 9. Discussion and Conclusions

EBV-associated natural killer (NK)/T-cell LPD is characterized by the proliferation of EBV-infected NK or T cells and exhibits varying clinical manifestations and prognoses. EBV-positive NK/T-cell lymphoproliferative disorders exhibit a notable geographic preference for East Asian and indigenous Latin American populations. This pattern is due to a complex interaction of factors: genetic vulnerabilities in these groups, such as risk HLA alleles [65] and immune response gene polymorphisms (e.g., the IL18RAP (rs13015714) and HLA-DRB1 (rs9271588)) [66], which weaken EBV immune surveillance [67]; endemic EBV strain variants with unique LMP1 oncogenic characteristics that are concentrated in regions with high incidence [68]. Early childhood EBV infections in these areas can lead to chronic active EBV and subsequent NK/T-cell lymphomagenesis, and environmental factors, such as pesticides and chemical solvents, can further influence disease risk [69]. Recent molecular profiling studies have revealed distinct genetic alterations and gene expression patterns associated with each condition, providing insights into their pathogenesis and potential therapeutic targets (Table 3). The aggressive subtypes, ENKTL and ANKL, exhibit a heterogeneous genomic landscape, yet they frequently present mutations in TP53, JAK/STAT, RNA helicase, and epigenetic dysregulation. Disruption of TP53 contributes to genomic instability and impaired cell-cycle control, while constitutive activation of the JAK/STAT pathway promotes unchecked proliferation, immune evasion, and resistance to apoptosis. Additional mutations involving RNA helicases and epigenetic modifiers further enhance transcriptional dysregulation and tumor adaptability. Together, these molecular abnormalities likely underpin the rapid disease progression, treatment resistance, and poor clinical outcomes observed in ENKTL and ANKL [65]. In contrast, CAEBV and HVLPD typically demonstrate a more restricted mutational burden, with fewer recurrent driver alterations involving TP53 and the JAK/STAT pathways. While EBV-driven signaling and host immune dysregulation play central roles in disease pathogenesis, the relative absence of widespread genomic instability and strong oncogenic driver mutations likely contributes to their more chronic clinical course, particularly in early stages. These molecular distinctions provide a biologic explanation for the marked differences in clinical behavior across EBV-associated T/NK-cell lymphoproliferative disorders and underscore the importance of genomic context in disease classification and risk stratification. The treatment of EBV-positive NK/T-cell lymphoproliferative disorders faces several critical challenges, including disease heterogeneity, lack of standardized therapies, high relapse rates, and limited curative options outside of allogeneic hematopoietic stem cell transplantation (HSCT). NK/T-cell lymphomas commonly exhibit a multidrug resistance phenotype, which renders anthracycline-based chemotherapy regimens ineffective [70]. Consequently, treatment strategies rely on non-anthracycline regimens, most often incorporating L-asparaginase. However, asparaginase-based therapies are associated with substantial toxicity, particularly in older patients and in those with impaired renal function, necessitating careful patient selection and dose modification [71]. Immune checkpoint inhibitors (ICIs), particularly PD-1 inhibitors, have demonstrated high response rates in relapsed or refractory EBV-positive NK/T-cell LPDs, with reported overall response rates of 60–100% and complete response rates of 30–70%, and are now considered preferred therapeutic options [72]. However, use of ICIs before allogeneic hematopoietic stem cell transplantation (HSCT) was associated with increased transplant-related mortality and a heightened risk of severe, hyperacute graft-versus-host disease, warranting careful treatment sequencing. In contrast, EBV-specific cytotoxic T-cell therapy has shown more modest efficacy (approximately 50% overall response and 30% complete response), likely reflecting the type II EBV latency program characteristic of EBV NK/T-cell LPDs, which limits expression of immunogenic viral antigens [70,72]. Additionally, the widespread application of EBV-specific T-cell therapy is constrained by substantial manufacturing challenges, including failure rates of up to 32%. Epigenetic dysregulation is common in mature T-cell and NK-cell lymphomas, supporting the use of epigenetic therapies, such as histone deacetylase inhibitors, which have shown encouraging activity in clinical trials [73,74]. In parallel, novel strategies, including cellular and adoptive immunotherapies, are actively under investigation. For chronic conditions, such as CAEBV and HVLPD, HSCT remains the sole curative option, potentially due to the involvement of HSCs. The observations lend credence to a model suggesting that all EBV+ NK/T-cell LPDs exist along a biological continuum of viral oncogenesis. In this model, a chronic EBV infection in a susceptible individual gradually accumulates oncogenic changes, with the clinical presentation being influenced by the host’s genetic and immune factors, as well as environmental co-factors [2]. This perspective connects indolent cutaneous EBV LPDs and aggressive NK/T-cell lymphomas through common EBV-driven mechanisms and the progressive somatic mutations occurring within the infected cell clone. Further research is needed to elucidate the molecular mechanisms driving these disorders and to develop more effective treatment strategies.

## Figures and Tables

**Table 1.** EBV NK/T-cell lymphoproliferative disorders.

EBV positive NK/T lymphoproliferative disorders more common in adulthood
• Extranodal NK/T-cell lymphoma (nasal type)
• Aggressive NK cell leukemia
• Primary nodal EBV-positive T/NK cell lymphoma (EBV-positive nodal T- and NK-cell lymphoma)*
EBV-positive T/NK lymphoproliferative disorders of childhood
•Hydroa vacciniforme lymphoproliferative disorder
- Classic
- Systemic
• Severe mosquito bite allergy
• Chronic active EBV disease, (systemic (T-cell and NK-cell phenotype)
•Systemic EBV-positive T-cell lymphoma of childhood

EBV denotes Epstein–Barr virus; the 2022 International consensus classification (ICC) terminology was provided within the parentheses, and the sign * denotes the provisional classification by ICC.

**Table 2.** The molecular subtypes of extranodal NK/T-cell lymphoma, nasal and non-nasal types.

Feature	TSIM (Tumor Suppressor /Immune Modulator)	MB (MGA-BRDT, MYC-Related)	HEA (HDAC9-EP300-ARID1A)
Key Genetic Alterations	*TP53* mutation, *JAK2, STAT3/5A/5B* mutations/amplifications, 9p24.1 amplification (*PD-L1/PD-L2, JAK2*), 6q21 deletion	*MGA* mutation, 1p22.1/*BRDT* LOH)	*HDAC9*, *EP300*, *ARID1A* mutations
Major Oncogenic Pathways	JAK-STAT pathway activation, PD-L1/PD-L2 overexpression (immune evasion)	MYC-driven oncogenesis, activation of *MAPK, NOTCH, WNT* pathways	NF-κB pathway activation, dysregulated TCR signaling pathway
EBV Characteristics	Latency II EBV (EBNA1, LMP1/2, EBERs), high BALF3 expression, EBV type 1	Latency I EBV (EBNA1+, LMP1–,EBER), low/absent LMP1 expression	Latency II EBV, high BNRF1 expression, lytic reactivation signatures
Transcriptomic/Epigenetic Features	Highest NK-cell gene expression signature, frequent *DDX3X* mutations	Mixed NK/T gene expression signature (leaning T-cell), high *MYC* expression, fewer *DDX3X* mutations	Highest T-cell gene signature, low NK marker expression, lowest *DDX3X* mutations, overexpression of DAXX
Cell-of-Origin	NK-cell origin	T-cell origin	T-cell origin
Clinical Outcomes	Intermediate prognosis (~79.1% 3-year overall survival)	Worst prognosis (~38.5% 3-year overall survival)	Best prognosis (~91.7% 3-year overall survival)
Potential Targeted Therapy	PD-1 or JAK inhibitors	MYC-targeted therapy	HDAC inhibitors

**Table 3.** Common genetic alterations observed in EBV-positive NK/T-cell lymphoproliferative disorders.

Disorder	Tumor Suppressors	Epigenetic /Chromatin Modifier Mutations	JAK/STAT Mutations	RNA Helicase	Copy Number Alterations	Pathway Activation
ENKTL	TP53 MGA,	BCOR, KMT2D, ARID1A, EP300	JAK2, STAT3, STAT5A, STAT5B	DDX3X	6q21 (deletion) (most common) 9p24.1 (gains)	JAK/STAT MYC NFkB
ANKL	TP53	TET2, KMT2D, CREBBP	JAK3, STAT3, STAT5B	DDX3X	7p (loss), 17p(loss) and 1q (gains) more common 6q loss less frequent	JAK/STAT RAS/MAPK pathways, and immune checkpoint molecules
ENTNKL		TET2 (64%)	STAT3 (19%)	DDX3X (20%)	14q11.2 loss (most frequent) Gain of 6p22.1	PI3K signaling (with frequent PIK3D (33%) mutations) IL6-JAK/STAT, cell cycle, genomic instability, PDL1 upregulation, interferon- α/γ response, NF-κB pathways
CAEBV (T/NK-type)	ATM (2.5%)	KMT2D (5%), BCOR/BCORL1 (3.8%), TET2 (2.5%), KMD6A (2.5%)	-	DDX3X (17.5%)	EBV intragenic deletions	cytokine signaling, NK/T-cell activity
HVLPD	ATM (14.3%), BARD1 (10.7%), MLH1 (10.7%)	KMT2D (28%), CREBBP (21.4%), BCOR (14.3%).	STAT3 (7%)	DDX3X (3%)	17p12/MAP2K4 (50%), 22q11./ SMARCB1 (36%), 16q24.3/ FANCA (36%), 12q13.12/ KMT2D (22.5%), and 6q21/PRDM1 (14.2%)	cytokine signaling, NK/T-cell activity and JAK signaling
SMBA	–	–	–	–	–	EBV-encoded gene expression, BZLF1
SCTCL		22q11.2 deletion/translocation		–	no defining recurrent abnormalities Once showed (1;22)(p22;q11.2) (22q11.2 locus)	Similar to ENKTL with overexpression of p53, survivin and EZH2.

ENKTL, extranodal NK/T-cell lymphoma; ANKL, aggressive NK cell lymphoma; ENTNKL, EBV-positive nodal T- and NK-cell lymphoma; CAEBV, Chronic active EBV disease (systemic NK/T type); SCTCL, systemic EBV-positive T-cell lymphoma of childhood

## Data Availability

No datasets were generated in this manuscript.
